# Lateral closing wedge high‐tibial osteotomy is a long‐lasting option for patients under the age of 55 with medial compartment osteoarthritis

**DOI:** 10.1002/jeo2.70040

**Published:** 2024-10-16

**Authors:** Ahmed Mahmoud, Bashirr Garba, Tim McMeniman, Brett Collins, Peter McMeniman, Peter Myers

**Affiliations:** ^1^ Brisbane Orthopaedic and Sports Medicine Center Brisbane Queensland Australia; ^2^ Department of Medicine The University of Queensland Brisbane Queensland Australia; ^3^ Department of Medicine Griffith University Brisbane Queensland Australia

**Keywords:** alignment, closing wedge, high tibial osteotomy, knee, osteoarthritis

## Abstract

**Purpose:**

Assess the survival of the closing wedge high tibial osteotomy (CWHTO) with failure defined as progression to total knee arthroplasty (TKA) and perioperative complications.

**Methods:**

Patients undergoing CWHTO in a single centre were included in this study. The patient's demographics, operative data and patient‐reported outcome measures were collected from the medical records. The outcomes assessed were progression to TKA, complications and patient‐reported outcome measures. The Australian joint registry was used to assess which patients progressed to TKA. A binary logistics regression is used to determine if any of the collected factors increase the likelihood of conversion to arthroplasty. Survival analysis is conducted using a Kaplan–Meier survivorship analysis with failure defined as progression to TKA.

**Results:**

Three hundred and fifty‐four (244 males and 110 females) patients were included in the study. The average age of the group was 51 years with an average follow‐up of 18 years. Patients under the age of 55 had a lower rate of progression to TKA. At 15 years, the rate of progression to TKA was 64% and 85% for those under the age of 55 and over 55, respectively. The complication rate was 6% without any peroneal nerve palsies.

**Conclusion:**

CWHTO is a good surgical option particularly when indicated in patients under the age of 55. Additionally, this technique results in a low overall complication rate with an absence of the often‐feared complication of peroneal nerve palsy.

**Level of Evidence:**

Level III, Retrospective study.

**Abbreviations**
95% CI: 95% confidence interval
ACLR: anterior cruciate ligament reconstruction
AOANJRR: Australian Orthopaedic Association National Joint Replacement Registry
CWHTO: closing wedge high tibial osteotomy
DVT: deep vein thrombosis
HTO: high tibial osteotomy
IKDC: International Knee Documentation Committee
MOWHTO: medial opening wedge high tibial osteotomy
OA: osteoarthritis
OCS: outerbridge cartilage score
PE: pulmonary embolus
PFJ: patellofemoral joint
PROMs: patient reported outcome measures
SD: standard deviation
TFJ: tibiofibular joint
TKA: total knee arthroplasty

## INTRODUCTION

Lateral closing wedge high tibial osteotomy (CWHTO) is a well‐accepted procedure for managing isolated medial compartment osteoarthritis (OA) and delaying or avoiding the need for a total knee arthroplasty (TKA). The goal is to correct the mechanical weight‐bearing axis and thus to relieve symptoms, delay the progression of OA in the medial compartment and potentially incite conditions for a spontaneous regeneration of cartilage [14, 23, 28, 41, 42]. A small degree of over correction is desirable [27, 30].

The Australian Orthopaedic Association National Joint Replacement Registry (AOANJRR) reports revision rates for TKA at 10 years to be more than seven times higher in patients under the age of 55 [3]. The maintenance of the native knee structures in CWHTO provides an alternative to TKA in patients engaging in high‐impact activity wherein significant loading on a prosthetic knee could contribute to a poor prognosis [15].

The short to mid‐term survival of the CWHTO procedure is well established in the literature. A steady decline in survival between 5 and 10 years is described in many studies, suggesting a similar decline in survival in the long term. However, limited data exist on the long‐term survival of CWHTO extending past 15 years. There has been a trend away from CWHTO due concerns about the perceived high rate of common peroneal nerve palsy and the technical demand of the operation [2, 30, 45, 51, 53]. The procedure requires careful attention to preoperative planning, soft tissue management, osteotomy technique and postoperative rehabilitation.

The aim of this study is to review a large cohort of patients following CWHTO from a single centre with long‐term follow‐up. Survival of the CWHTO is the primary outcome assessed with failure defined as progression to TKA. The second aim of this study is to document perioperative complications. We hypothesise that most patients will progress to TKA by 20 years and that patients under the age of 55 will have a lower rate to progression to TKA.

## MATERIALS AND METHODS

Ethical approval for the acquisition and use of patient data was obtained from the Mater Misericordiae Limited Human Research Ethics Committee (HREC/MML/49345.V4). Patients undergoing CWHTO from a single centre between June 1989 and October 2003 were initially retrospectively reviewed in 2004. The patients included were those who underwent an isolated CWHTO or a CWHTO with other concomitant procedures such as ligament reconstructions. Patients were excluded if they declined to participate in the study or were under the age of 18. This same cohort has been followed in 2019 and 2020 for this study. The patients were treated by three surgeons at the centre and received standardised pre‐, intra‐ and postoperative care.

The indication for CWHTO was symptomatic medial compartment osteoarthritis with varus deformity in a patient with a stable or stabilisable knee. Contra‐indications were poor range of motion, defined as knee flexion less than 100° or fixed flexion deformity of >10°. Relative contraindications were significant chondral changes (outerbridge grade >3) in the lateral compartment or significant lateral meniscal damage seen at the time of surgery. The desired angle of correction was determined using hip‐ankle standing films and the Miniaci technique [35]. The desired mechanical axis was taken through the lateral tibial spine or on the downslope of this. This technique aimed to overcorrect the natural valgus alignment by 2^o^.

### Operative technique

With a tourniquet applied, the knee is positioned at 90° on a foot roll. Arthroscopy was performed at the time of surgery in 289 (81.6%) cases to assess the individual compartments and thus to confirm suitability to proceed. An 8‐to‐10‐cm longitudinal incision is made midway between the fibular head and tibial tuberosity, the deep fascia is exposed. A curved fascial incision is made beginning anteriorly on the fibular head extending proximally over the tibiofibular joint (TFJ) before moving anteriorly along the tibial flare curving around the attachment of tibialis anterior and ending distally adjacent to the tibial tuberosity. The anterolateral surface of the tibia and the anterior aspect of the fibular head is exposed, and the muscles reflected inferiorly. The periosteum is released anteriorly and proximally and a Cobb's periosteal elevator is used to expose anteriorly reflecting the patellar tendon anteriorly.

**Figure 1:**
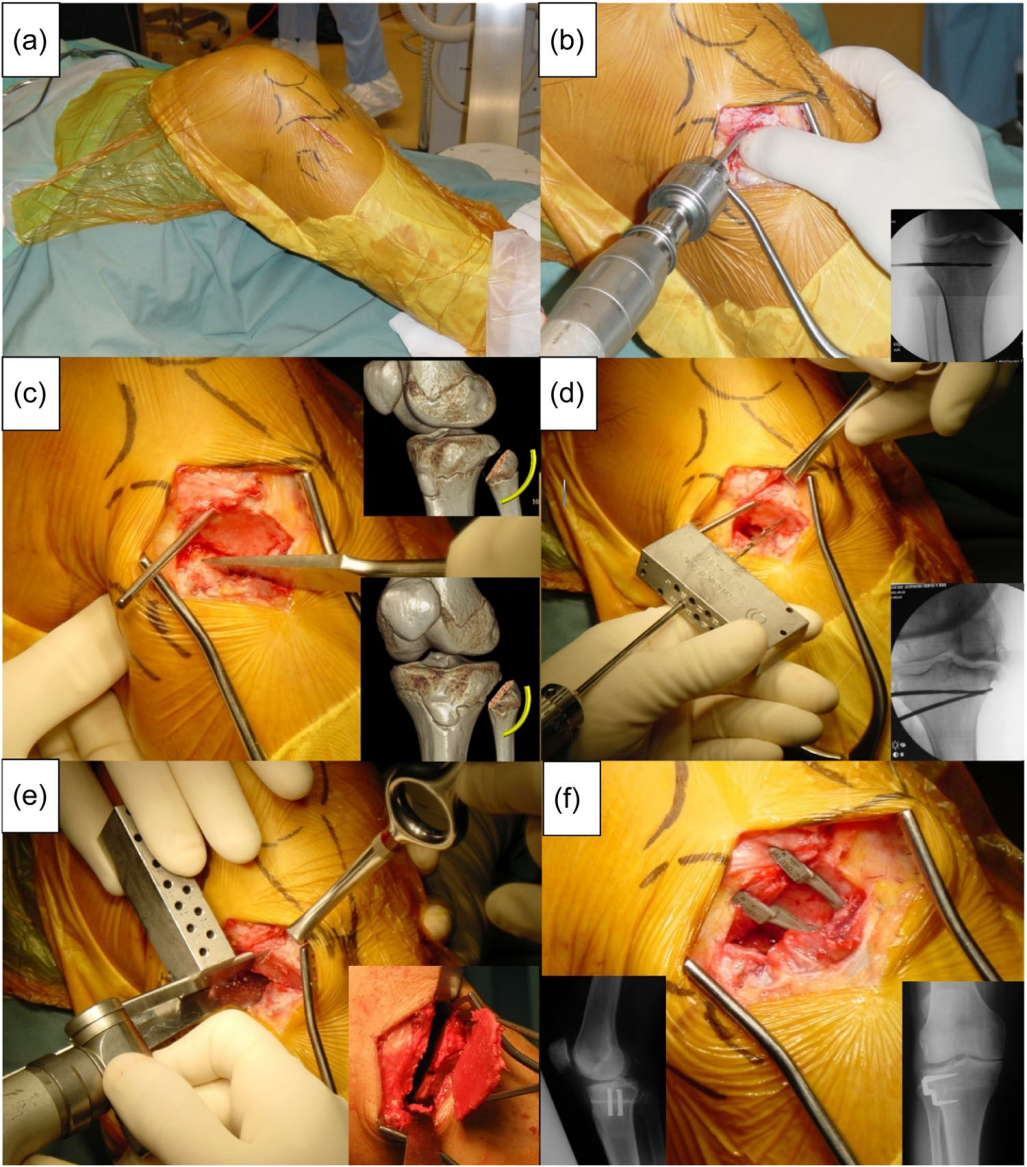
The surgical technique of closing wedge high tibial osteotomy (CWHTO). (a) Positioning. (b) Approach and first wire. (c) Fibula osteotomy to protect the Common Peroneal nerve. (d) Second wire placement. (e) Performance of osteotomy using saw. (f) Correction and insertion of staples.

Using a number 10 blade, the TFJ is incised and the plane of the joint determined. A 1.5 cm wide osteotome is used to release the TFJ capsule, the osteotome must be kept in the plane of the joint and not directed postero‐medially towards neurovascular structures. Following TFJ release, only the antero‐medial aspect of the fibular head is excised using a fine saw followed by an osteotome. This osteotomy is begun around 4‐mm inferior to the TFJ and directed slightly anteriorly (towards the styloid process) and rotated internally to take more bone inferiorly than proximally. The peroneal nerve is palpable posterior to the neck of the fibular and with the knee flexed at 90° the fibular head osteotomy is directed away from the nerve. The fibular head can then be levered posteriorly protecting the peroneal nerve (Figure 1).

Under direct vision, the posterior periosteum of the proximal tibia is then elevated using the Cobb's elevator. Hohman retractors are placed posteriorly and anteriorly exposing the anterior, lateral and posterior cortices. Under fluoroscopic control, a drill bit is placed from lateral to medial parallel to the tibiofemoral joint to 1 cm from the medial cortex. A second drill bit is placed at the chosen wedge angle using a jig system and confirmed fluoroscopically. An oscillating saw is used to make the tibial cuts using the drill bits as ‘cutting guides’. The medial cortex is not breached by the saw. The proximal cut is done first parallel to the tibial slope ensuring that the posterior cortex is cut in the ‘curve’ of the tibia safely away from the popliteal artery. The distal cut is made parallel to the proximal cut (from a lateral perspective) using the distal drill bit as the guide. The wedge is grasped and removed in one piece. An angled curette is used to ensure that the anterior and posterior cortices are clear, with no bone remaining that may impinge on the patellar ligament or obstruct closure of the osteotomy. The osteotomy is closed, usually with a soft cracking sound. An osteotome can be used to gently extend the anterior and posterior cortical releases if there is difficulty with the closure. Fixation is then performed according to surgeon preference as stated in the results section.

A suction drain is placed posteriorly to minimise risk of compartment syndrome, this is removed after 24 h. The anterior compartment is closed and the wound closed with absorbable sutures.

### Postoperative management

The knee is placed in an extension brace. The patient mobilises touch weight bearing on the operated leg. At 2 weeks, dressings are removed and weight bearing is begun and progressed at 25% of body weight per week with physiotherapy supervision. Gentle range of motion exercises are also begun progressing to 90° at six weeks and 120° by 12 weeks. The brace is discarded when quadriceps control is regained. The patient progresses to independent mobility by around 8–10 weeks from surgery. At 6 weeks, a radiograph is obtained to confirm adequate bone healing. Structured rehabilitation continues until 4 months postoperatively and home exercises are advised for a further 3 months.

### Data collection

Demographics, complications, living status and later conversion to arthroplasty data were collected from the medical records and by contacting patients. Also, in 2021, the patient's details were cross‐referenced with data from the Australian Orthopaedic Association National Joint Replacement Registry (AOANJRR) to capture all patients who had progressed to arthroplasty.

Patient‐reported outcome measures (PROMs) were collected at a minimum of a year postoperatively for the initial study in 2004. The PROMs used were the Lysholm Knee Scoring Scale, Oxford Knee Score, Tegner Activity Level Scale and International Knee Documentation Committee to comprehensively assess overall patient function. Also, patients were asked their level of satisfaction with the procedure and whether they had undergone further surgical procedures on the knee. The same questionnaire was issued in 2020 to all participants who had not progress to TKA. Complications were considered early if they occurred during hospitalisation and late if they occurred after discharge.

### Statistical analysis

Statistical analysis was performed using the SPSS v25 program. The patient demographics are demonstrated using descriptive statistics in means and standard deviations. A binary logistics regression is used to determine if any of the collected factors increase the likelihood of conversion to arthroplasty. These factors include age, gender, wedge angle, cartilage status in the lateral and patellofemoral compartment, preoperative fixed flexion deformity, or concomitant ACLR. Survival analysis is conducted using a Kaplan–Meier survivorship analysis with failure defined as progression to TKA. *p*‐values < 0.05 were considered statistically significant.

## RESULTS

**Figure 2:**
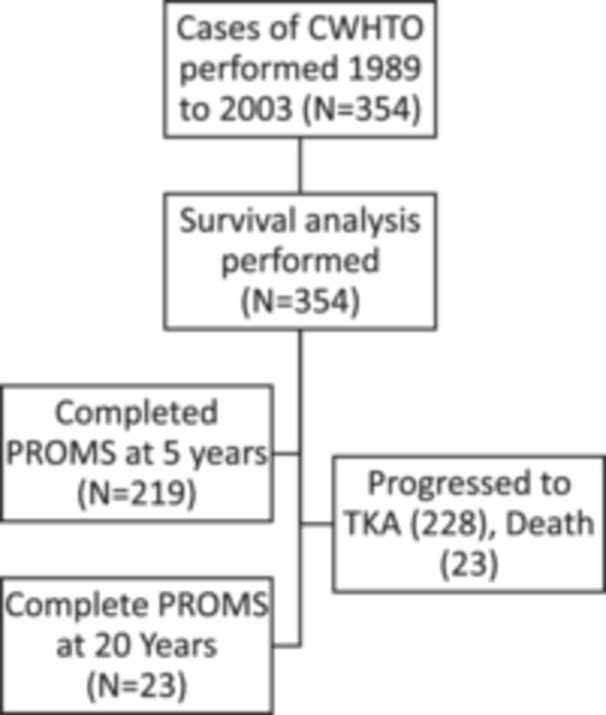
Flow chart demonstrating patients included in the closing wedge high tibial osteotomy (CWHTO) in different phases of the study. PROMs, patient reported outcome measures; TKA, total knee arthroplasty.

**Figure 3:**
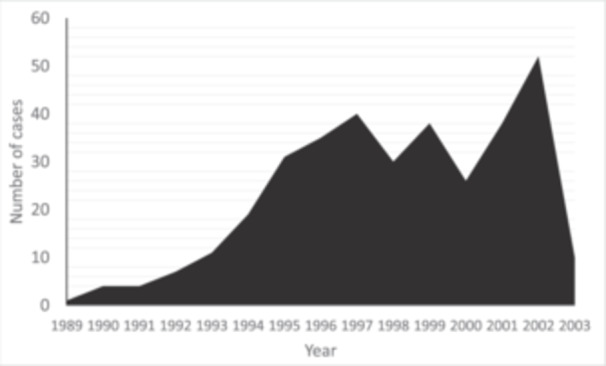
The number of yearly closing wedge high tibial osteotomy performed over the period of the study.

A total of 306 patients were included in the study, undergoing 354 CWHTOs (Figures 2 and 3). Surgeon 1 performed 183 (51.7%) surgeries, surgeon 2 performed 159 (44.9%) and surgeon 3 performed 12 (3.4%). Demographic data are displayed in Table 1. Bilateral surgeries were performed in 48 patients, 46 patients underwent staged procedures, and two patients underwent simultaneous bilateral procedures. Two hundred and eight cases (60%) were under the age of 55 at the time of surgery. Fixation was achieved using one to two staples in 231 (65.3%) cases; a first step device (Orthocare Pty Ltd) in 90 (25.4%) cases, and a plate and screws in 14 (4%) cases. Fixation was not recorded in 19 (5.3%) cases.

**Table 1:** Subject demographics.

	Mean (SD)	Percentage
Age	51.6 (8.5)	
Wedge angle (degrees)	9 (2.7)	
Follow‐up (years)	18 (6.4)	
Bilateral CWHTO		13.5
Gender		69 Males
Side		54.6 Right
Concurrent ACLR		8
Lateral compartment OCS		
Grade 0–1		77.9
Grade 2		9.2
Grade 3		1.5
Not assessed		21.4
PFJ OCS		
Grade 0–1		20.6
Grade 2		27.5
Grade 3		18.5
Grade 4		0.6
Not assessed		32.9

Abbreviations: ACLR, anterior cruciate ligament reconstruction; CWHTO, closing wedge high tibial osteotomy; OCS, outerbridge cartilage score; PFJ, patellofemoral joint, SD, standard deviation.

### Survival

**Figure 4:**
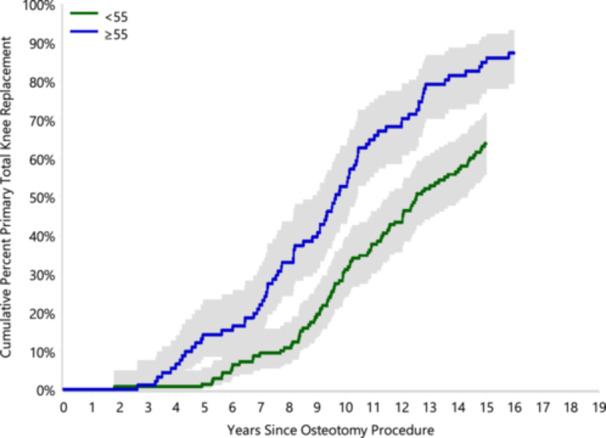
Cumulative percent revision of primary total knee replacement after closing wedge high tibial osteotomy by age.

The AOANJRR reported that 228 (64%) patients from our cohort had progressed to TKA. Twenty‐three patients had died from unrelated causes. The cumulative incidence curve compares the cohort by age (above 55 years and below 55 years old) of conversion to TKA (Figure 4 and Table 2). The rate of progression to TKA by 15 years post‐CWHTO for patient under the age of 55 is 63.8%, while it is 84.9% for those over the age of 55. The binary logistic regression analysis found that age was the only significant predictor of conversion to TKA. The estimated odd ratio favoured an increase of 5% (odd ratio: 1.05, 95% confident interval [CI]: 1.005–1.098, *p*‐value = 0.028) for conversion to TKA for every year increase in age. The rate of revision TKA was 3.5% (*N* = 8) for the cohort of patients who underwent TKA postosteotomy.

**Table 2:** Yearly cumulative percent primary total knee replacement after osteotomy.

Cumulative percent progression to total knee arthroplasty	5 years	10 years	15 years
<55 years	1.4 (0.4, 5.7)	31.2 (24.2, 39.6)	63.8 (55.8, 71.7)
≥55 years	14.3 (8.6, 23.3)	52.7 (42.9, 63.3)	84.9 (76.7, 91.4)

### Clinical outcomes

A total of 219 (69.3% of the total patients) patients responded to the first issue of the PROMs postoperatively. Mean follow‐up duration for PROMs was 5.5 years (SD 3.6). Eighty‐three per cent of patients were very satisfied or satisfied with the outcome of the procedure. Table 3 demonstrates the results of the postoperatively PROMs by age. Although the Oxford score was significantly different between the two age groups. The Tegner score indicates that patients were mostly able to participate in light recreational sports such as cycling or swimming.

**Table 3:** Patient reported outcome measures by age groups.

	<55 (SD*) (*N* = 156)	>55 (SD) (*N* = 74)	Score interpretation	*T*‐test (CI and *p*‐value)
Lysholm	69.3 (19.5)	65.3 (20.9)	Fair	−1.5 to 9.6, 0.155
IKDC	59.5 (18)	54.8 (16)		−0.1 to 9.6, 0.056
Oxford	18 (12.4)	14 (14.5)		1.2 to 6.9, 0.006
Tegner	3.7 (1.5)	3.3 (1.4)		−0.04 to 0.8, 0.075

Abbreviations: 95% CI, confidence interval; IKDC, International Knee Documentation Committee; SD, standard deviation.

**Figure 5:**
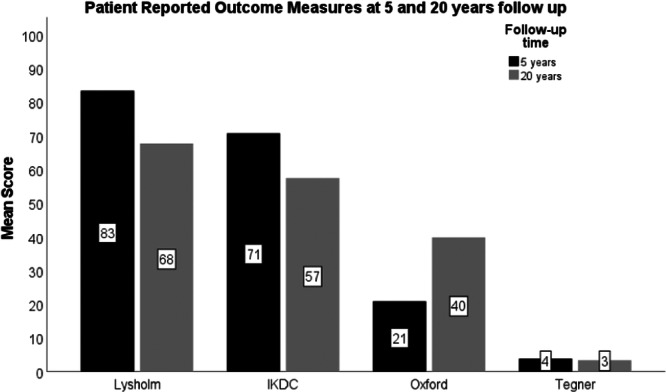
Comparison of patient reported outcome measures at 5 and 20 years postoperatively.

At 20 years follow‐up, 23 patients completed the PROMs as there were very few patients who were alive and did not progress to TKA. The mean scores in the same patients that completed the follow‐up at 5 years and 20 years are demonstrated in Figure 5. As expected, the Lysholm, IKDC and Tegner score decreased, however, the Oxford score improved.

## COMPLICATIONS

There were no intra‐operative complications. The overall complication rate was 5.9% (Table 4). Early complications (in hospital) occurred in seven (2.0%) patients. There were no cases of deaths, peroneal nerve injury, vascular injury, loss of correction, or compartment syndrome. In one patient, it was recognised after surgery that the preoperative alignment X‐rays had not been done weight bearing. This patient underwent a revision of the procedure during the same hospitalisation.

**Table 4:** The complication profile after closing wedge high tibial osteotomy.

	Early^a^ (*N* = 7, 2%)	Late^a^ (*N* = 14, 4%)
Infection	1	0
Revision of osteotomy	1	0
Deep vein thrombosis	3	5
Pulmonary embolism	2	2
Delayed union		7

^a^ Early: During hospitalisation. Late: After discharge

## DISCUSSION

Our study presents a long‐term retrospective review of CWHTO outcomes in a large cohort of patients presenting to a single centre. The study shows that the CWHTO procedure had better survivorship in those under the age of 55 across all time points. Most patients over the age of 55 progressed to total knee replacement by 15 years of follow‐up. Our results at a follow‐up of 5, 10 and 15 years are consistent with other published studies [2, 6, 9, 11, 15, 18, 25, 30, 31, 36, 37, 46, 47, 50]. Similarly to our study, Constantin et al. found that age >55 at the time of CWHTO was associated with a higher rate of progression to TKA.

Several studies have investigated the influence of preoperative factors on HTO survival. These have included age, sex, weight/body mass index, severity of OA using Ahlbach grade, preoperative varus angulation, lateral thrust, presence of flexion contracture, preoperative range of motion, ACL deficiency and previous surgeries [1, 2, 6, 7, 11, 12, 15, 18, 24, 25, 36, 46, 50]. Our study supports that a higher age at time of CWHTO increases the risk of progression towards a TKA.

Whether or not age should be a contraindication to HTO is unclear with the upper limit recommended ranging between 50 and 65 years [15, 18, 25, 36, 48]. Trieb et al. concluded that HTO is not suitable for patients over 65 after comparing the risk of progression to TKA between patients under or over the age of 65 [48]. Other authors have stressed the importance of level of activity over chronological age [18, 31]. In line with several previous reports [15, 25, 36], our study demonstrated considerably better survival in patients under the age of 55. Our results indicate that 52% of patients >55 years of age progress to TKA by 10 years and 85% by 15 years.

Intra‐ and postoperative factors that may influence survival include angle of correction, fixation method and loss of angle of correction over time [1, 2, 6, 7, 11, 15, 20, 30, 36, 37, 43, 46]. Achieving an appropriate degree of valgus angulation is the most widely cited factor contributing to long‐term survival of the procedure. The desirable range is variable, with many authors aiming for overcorrection to between 6 and 15° of anatomic valgus [1, 7, 30, 46]. In addition to offloading the medial compartment and potentially allowing for regeneration of the damaged articular surface, an adequate angle of overcorrection serves to prolong the time to loss of the angle of correction [10, 55]. Coventry suggested 8° as a minimum angle of correction without prescribing an upper limit [11], however, cosmetic concerns have been raised with angulation above 10° [25, 26, 46]. Furthermore, extensive overcorrection has been linked to development of degenerative changes in the lateral compartment [22]. Reliability in achieving the desired angle of correction is therefore paramount to long‐term success. Opening wedge and closing wedge osteotomies have recently been found to have different impacts on the proximal tibia correction [29]. Kim et al. showed that CWHTO can be associated with a higher effectiveness in intra‐articular correction compared to the opening wedge technique. Our approach aimed to overcorrect the natural valgus alignment by 2°; we considered it important to consider the natural alignment of the contra‐lateral limb when determining wedge size which called for some adjustments to this goal. In this study, we have reported a standardised reproducible surgical technique using an accurate and simple jigging system. The osteotomy technique described is suitable for other jig systems. Using this technique, we achieved a suitable mean angle of correction of 9° across all cases.

Aside from difficulties in achieving and maintaining a suitable angle of correction, the complications of the HTO procedure include patellar height changes, hinge fractures, delayed union, nonunion, removal of hardware, compartment syndrome, infection, thromboembolic disease and nerve palsies [33, 45, 49, 51]. The rate of complications varies significantly between studies. In 2006, Flecher et al. reported a complication rate of 3.3% among 372 osteotomies [15]. In the same year, Papachristou et al reported a complication rate of 16% after 44 osteotomies [37]. Studies in the lower end of the range typically achieve complication rates below 10%; [15, 25, 30] studies in the higher end report rates above 30% in some instances [36, 50]. This variation may be attributed to differences in patient selection, surgical technique and postoperative care regimens. With the exception of patellar height changes, the rate and type of complications encountered do not appear to be specific to the closing wedge approach, occurring in similar rates in the medial opening wedge high tibial osteotomy (MOWHTO) procedure as well [44]. We present a low overall complication rate of 6% with no patients suffering any long‐term sequelae and notably no incidence of peroneal nerve palsy, patella baja, or nonunion.

The occurrence of peroneal nerve palsies is the most noted disadvantage of the CWHTO procedure when comparing it to MOWHTO. Some articles point to an incidence of palsy as high as 20% as described by Wootton et al [51, 53]. It should be noted that, in 11 of the 21 cases of peroneal nerve palsy described by Wootton, the impairment was only transient in nature [53], this is consistent with other studies in the literature [2, 30, 45]. Furthermore, in meta‐analysis of clinical outcomes, Smith et al. failed to find a significant difference in the incidence of common peroneal nerve palsy between CWHTO and MOWHTO [44]. The incidence of peroneal nerve palsies can likely be largely attributed to surgical technique. Disrupting the strutting effect of the fibula is crucial in obtaining and maintaining correction of the osteotomy. However, it is this step which is a major concern for potential peroneal nerve injury. In a cadaveric study, Kirgis and Albrecht described the high‐risk zone for causing motor nerve injury to be 68–153 mm distal to the fibular head, recommending against mid‐shaft fibular osteotomy [16]. This recommendation is supported by Wootton, who divided the fibula into four zones and found 80% of peroneal nerve palsies occurred in fibular osteotomies performed in zone III, located 80–150 mm distal to the fibular head, but no palsies occurred in zones I or IV (fibular head osteotomy or 160 mm distal to fibular head, respectively) [53]. Furthermore, in an examination of the aetiological factors contributing to nerve injury, Bauer et al concluded that, in addition to direct trauma, a measure of compartment syndrome and stretching injury may occur during the osteotomy and fixation manoeuvres on a nerve that is sensitised to damage secondary to intraoperative ischemia [5]. As a result, they recommended the use of a tibial resection jig system to minimise traumatic manoeuvres. Development of elevated compartment pressures postoperatively can be mitigated by placement of a suction drain as demonstrated by Gibson et al [17]. Our technique involves disrupting the TFJ in the plane of the joint and excising a small amount of the anteromedial aspect of the fibular head in a manner which does not endanger the nerve. In addition, careful placement of retractors, the employment of a jig system, and placement of a posterior drain all mitigate the risk of peroneal nerve injury. We believe adherence to careful surgical technique is responsible for the absence of neurovascular complications amongst our large cohort.

In most cases, a failed tibial osteotomy occurs due to progression of OA, necessitating the need for a TKA. Thus, it is pertinent to consider the implications the HTO procedure has on subsequent TKA outcomes. Potential issues raised can be divided into operative and postoperative. Operative concerns include anatomical deformities of the proximal tibial compartment (including alterations to the posterior tibial slope angle), bone stock loss, a damaged posterior cruciate ligament footprint, peg positioning, increase operative time, and difficulties achieving joint line height and coronal balance [8, 19, 49, 51, 52]. In the postoperative period, some authors have raised concerns over the incidence of extensor mechanism mal‐tracking, instability, limb malalignment aseptic loosening and increased revision rates [8, 39]. Certainly, this procedure can be more technically challenging than a primary TKA due to alterations in the anatomy, mechanical axis, increased incidence of preoperative malalignment, soft tissue scarring and the presence of hardware [19]. However, Kuwashima et al. noted that the difference in postoperative deformity of TKA after CWHTO or OWHTO was clinically irrelevant [32]. Despite the decreasing popularity of CWHTO over the years, it still has a role in the prevention or delaying the need of TKA without compromising future surgical options.

The initial clinical and survival data of TKA after HTO were concerning, however, more recent literature has shown no difference in outcomes. Parvizi et al cautioned higher rates of radiolucencies and poorer functional scores in post‐HTO TKA patients [39]. A higher revision rate was supported by Haslam et al.; however, no difference in clinical outcomes was observed [21]. More recent studies, including a systematic review by Ramappa et al., have demonstrated comparable survival, radiological, and clinical outcomes between primary and post‐HTO TKA patients [4, 8, 38, 40]. Xie et al. showed that a delayed TKA after HTO has better longer terms survival than TKA in a matched cohort [13]. Also, clinical outcomes of TKA following HTO were similar to primary TKA [54]. Our overall rate of revision TKA was 3.5% which is similar to reported data. Advances in surgical technique in both procedures may be responsible for closing the outcome gaps reported in older studies. Although technically challenging, the CWHTO demonstrates good survival in the long term with lack of negative impact of sex, amount of correction and associated procedures on survival. The technique in this article demonstrates a safe approach without the risk of common peroneal nerve palsy and nonunions. The CWHTO should remain as a valuable option for the young patient with medial compartment osteoarthritis.

### Limitations

This study is limited in part due to its retrospective nature. This single‐centre study afforded a high measure of consistency in the pre‐, intra‐ and postoperative management of patients at the potential cost of limiting the external validity of the results. Though the primary outcome of survival was well supported through clinical records and joint registry data, we recognise our conclusions would be strengthened by the addition of radiological data. Clinical outcomes were collected prospectively at two time points to increase the robustness of the results; however, we acknowledge this may introduce self‐selection bias to our results with a skew towards favourable outcomes. Additionally, like most studies of this nature, we defined survival as the progression to TKA which, from a patient's perspective, may not be an adequate measure of procedure success. This point is highlighted by Matthews et al. who defined survival as maintaining symptomatic relief with continued useful function and no further operation and reported only 50% useful function at 5 years [34]. Other limitations included one of the surgeons had a limited number of procedures (3.4%), different fixation methods were used and OCS was not assessed in a significant number of cases (lateral compartment 21.4% and patella‐femoral joint 32.9%).

## CONCLUSION

CWHTO is a good surgical option particularly when indicated in patients under the age of 55. Additionally, this technique results in a low overall complication rate with an absence of the often‐feared complication of peroneal nerve palsy.

## AUTHOR CONTRIBUTIONS

The authors Peter McMeniman and Peter Myers contributed by initiating the project, performing the interventions and in patient follow‐up. The authors Ahmed Mahmoud and Bashirr Garba performed the data collection, analysis and article write up. The Tim McMeniman and Brett Collins contributed to data collection, literature review and ethical approval. All authors read and approved the final manuscript. Open access publishing facilitated by $INSTITUTION, as part of the Wiley ‐ $INSTITUTION agreement via the Council of Australian University Librarians.

## CONFLICT OF INTEREST STATEMENT

The authors declare no conflict of interest.

## ETHICS STATEMENT

Ethical approval for the acquisition and use of patient data was obtained from the Mater Misericordiae Limited Human Research Ethics Committee (HREC/MML/49345.V4). The patients all consented to be part of the research project.

## Data Availability

The data sets generated and/or analysed during the current study are not publicly available due to patient confidentiality but are available from the corresponding author upon reasonable request. Patients consented to use of data in this project.
